# Epidemic evolutionarily stable strategies within an age-structured host population

**DOI:** 10.1073/pnas.2418170122

**Published:** 2025-03-18

**Authors:** Andreas Eilersen, Ottar N. Bjørnstad, Ruiyun Li, Sebastian J. Schreiber, Zeyuan Pei, Nils Chr. Stenseth

**Affiliations:** ^a^Theoretical Biology Group, Department of Environmental Systems Science, ETH Zürich, Zürich 8092, Switzerland; ^b^PandemiX Center, Department of Science and Environment, Roskilde University, Roskilde 4000, Denmark; ^c^Department of Entomology, Pennsylvania State University, University Park, PA 16802; ^d^Centre for Ecological and Evolutionary Synthesis, Department of Biosciences, Faculty of Mathematics and Natural Sciences, University of Oslo, Oslo 0371, Norway; ^e^Department of Epidemiology, School of Public Health, Nanjing Medical University, Nanjing 211166, Jiangsu, China; ^f^Jiangsu Center for Collaborative Innovation in Geographical Information Resource Development and Application, Nanjing University, Nanjing 211166, China; ^g^Department of Evolution and Ecology, University of California, Davis, CA 95616; ^h^Centre for Pandemics and One-Health Research, Sustainable Health Unit, Institute of Health and Society, Faculty of Medicine, University of Oslo, Oslo 0316, Norway; ^i^Vanke School of Public Health, Tsinghua University, Beijing 100084, China

**Keywords:** evolution, modelling, epidemiology, trade-off, demographics

## Abstract

To understand infectious disease dynamics, we need to understand the inextricably intertwined nature of the ecology and evolution of pathogens and hosts. Epidemiological dynamics of many infectious diseases have highlighted the importance of considering the demographics of the societies in which they spread, particularly with respect to age structure. In addition, the waves of the recent COVID-19 pandemic driven by variant replacements at an unprecedented speed show that it is vital to consider the evolutionary aspects. The classic trade-off theory of virulence addresses aspects of pathogen evolution, but here we explore in more detail the possibility of society-specific evolutionarily stable strategies (ESS) during an unfolding pandemic. Theory posits the existence under some conditions of an ESS representing the evolutionary endpoint of change. By using a demographically realistic model incorporating infection rates that vary with age, we outline which evolutionary scenarios are plausible. Focusing on the rate of infection and duration of infectivity, we ask whether an ESS exists, what characterizes it, and as a result which long-term public-health consequences may be expected. We demonstrate that the ESS of an evolving pathogen depends upon the background age-dependent frailty and mortality rates. Our findings shed important light on the plausible long-term trajectories of highly evolvable novel pathogens.

## Significance

Emerging pathogens are usually far from any phenotypical optimum in relation to their new hosts. The concept of evolutionarily stable strategies (ESS) may be helpful for predicting the evolution of such pathogens. The ESS represents a pathogen variant with an optimal phenotype, which cannot be outcompeted by invading mutants. We investigate the ESS for a pathogen in an age-structured population, assuming a trade-off between pathogen infectivity and the duration of disease analogous to the classic virulence–infectivity trade-off. We show how a population divided into age stages with different demographic parameters affects the evolution of the pathogen and shapes its traits. As many human pathogens cause a highly age-dependent disease burden, this provides important projections about the direction of evolution.

The fate of pandemics has long been considered in the field of epidemiology and evolutionary biology, and this topic has been greatly emphasized by the rapid variant replacement seen during the COVID-19 pandemic (1, 2). The epidemiological dynamics of any emerging pathogen in a new host face steep selective gradients—as seen for example with SARS-CoV-2—because the phenotype of the initial strain and early variants will typically be far from any evolutionary equilibrium. The reason for this is that emerging pathogens are not yet evolutionarily optimized for the human niche neither at the individual nor community level (3). Regarding pathogen genotype, evolution does not necessarily have any endpoint (4), but the phenotype may at some point reach an optimum for the new host. Historical evidence from a range of pathogens (5–7) provides case studies of how variants of intermediate virulence will often—though not always (8, 9)—win over virulent and avirulent variants in the long term. This was for example demonstrated in the seminal study by Fenner et al. (10) of novel introduction of myxomatosis in rabbits for biological control (11, 12). The long-term evolutionary equilibrium of the virulence, transmissibility, and disease duration of emerging pathogens has been subject to particular interest in evolutionary virology.

The virulence–infectivity trade-off was in the above cases a reduction in disease duration brought on by high virulence and death of hosts. In COVID-19 and other respiratory pathogens, there are also trade-offs. These are however more related to either hospital isolation or elicitation of strong immune responses leading to clearance, thus leading to a negative correlation between infectious period duration and viral replication. While not as commonly investigated as the virulence–infectivity trade-off, there is some precedent for assuming an infectivity–recovery rate trade-off affecting the evolution of pathogens (13, 14). Investigating how pathogen evolution toward an evolutionarily stable strategy (ESS) may modulate epidemic dynamics for such scenarios is therefore highly relevant.

Fifty years ago, mathematical studies of the fundamentals of evolutionary dynamics defined the idea of the ESS—the long-term likely outcome of ecoevolutionary dynamics (8, 9, 15). An ESS is a strategy where no mutant variant of a pathogen is able to invade and displace a specific final variant when in a stationary epidemiological state (16–19). It thus represents a potential evolutionary endpoint after pandemic emergence to endemicity. ESS conditions have been investigated in epidemiological and ecological systems (20). Additionally, an effect of population age structure on disease infectivity and virulence has been suggested in studies of many host–pathogen systems (21). Here, we elaborate on these fundamental ideas in the context of evolving pathogens in human populations to study how age structure may affect the evolutionary endpoint.

## Results

### The Demographic Epidemiology Model.

**Fig. 1.**
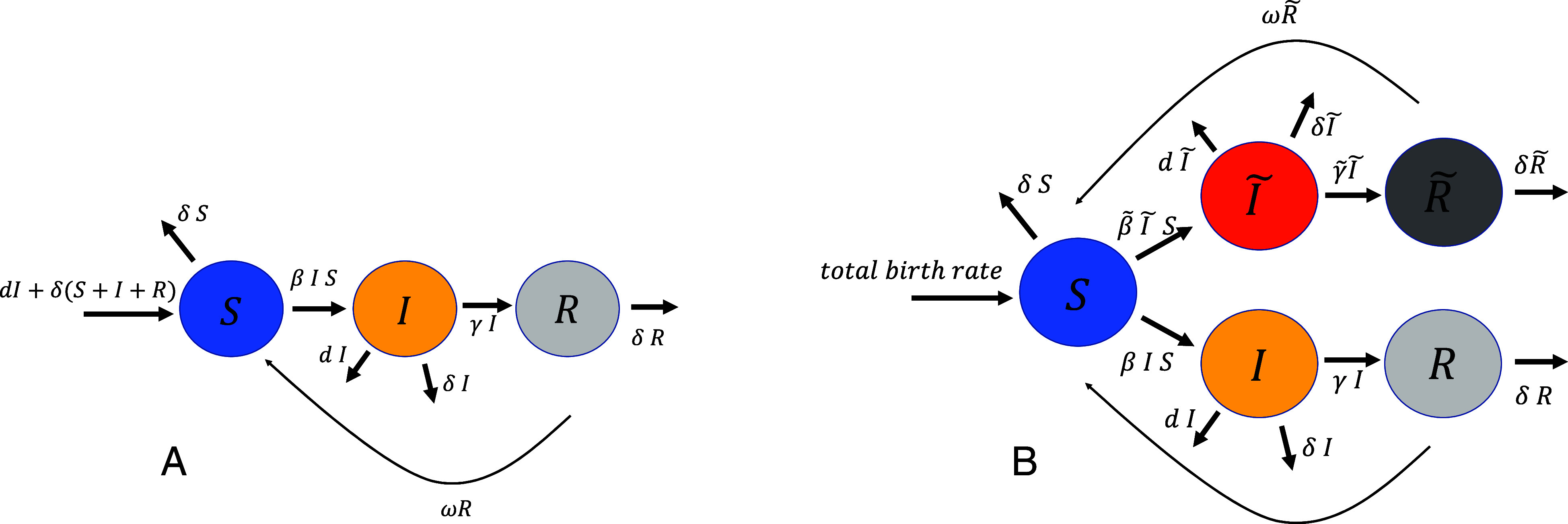
An illustration of the models and parameters. (*A*) The model with a single variant. (*B*) The extended model with a resident and a mutant variant. Each compartment shown in this illustration is further subdivided into *n* age stages.

Based on a previous model (22), we elaborate on evolutionary trajectories likely to unfold in an age-structured susceptible-infected-recovered-susceptible (SIRS) setting (Fig. 1*A*). The basic SIRS-based model partitions the host population into *n* age stages of susceptible (*S*), infected (*I*), and recovered (*R*) individuals. The initiation is a single strain (hereafter the “wild-type strain”). For age stages i≥2, the model is given as

[1] $$\begin{array}{c} \frac{dS_{i}}{\mathrm{dt}}=\underset{\mathrm{aging in}}{\underset{︸}{a_{i\;-\;1}S_{i-1}}}-\underset{\mathrm{aging out}}{\underset{︸}{a_{i}S_{i}}}-\underset{non-disease\;deaths}{\underset{︸}{\delta_{i}S_{i}}}\\ \;+\underset{\mathrm{loss of immunity}}{\underset{︸}{\omega R_{i}}}-\underset{\mathrm{infection}}{\underset{︸}{\sum_{j}\beta_{\mathrm{ij}}S_{i}I_{j}}}\\ \frac{dI_{i}}{\mathrm{dt}}=\underset{\mathrm{aging in}}{\underset{︸}{a_{i-1}I_{i-1}}}+\underset{\mathrm{infection}}{\underset{︸}{\sum_{j}\beta_{\mathrm{ij}}S_{i}I_{j}}}-\underset{\mathrm{aging out}}{\underset{︸}{a_{i}I_{i}}}\\ \;-\underset{\mathrm{recovery}}{\underset{︸}{\gamma_{i}I_{i}}}-\underset{\mathrm{disease deaths}}{\underset{︸}{d_{i}I_{i}}}-\underset{non-disease\;deaths}{\underset{︸}{\delta_{i}I_{i}}}\\ \frac{dR_{i}}{\mathrm{dt}}=\underset{\mathrm{aging in}}{\underset{︸}{a_{i-1}R_{i-1}}}+\underset{\mathrm{recovery}}{\underset{︸}{\gamma_{i}I_{i}}}-\underset{\mathrm{aging out}}{\underset{︸}{a_{i}R_{i}}}\\ \;-\underset{non-disease\;deaths}{\underset{︸}{\delta_{i}R_{i}}}-\underset{\mathrm{loss of immunity}}{\underset{︸}{\omega R_{i}}} \end{array}.$$

Here, $S_{i}$, $I_{i}$, and $R_{i}$ are susceptible, infected, and recovered compartments of the populations of age stage i, $a_{i}$ are the aging rates for age stage i allowing for age brackets of different lengths, $d_{i}$ and $\delta_{i}$ are the age-specific disease-induced and background mortality rates, respectively, $\gamma_{i}$ are the possibly age-dependent recovery rates, and ω is the rate of loss of immunity, which for simplicity we assume to be age-invariant. For n age stages, there will be $n^{2}$ rates $\beta_{\mathrm{ij}}$ of infection from age stage j to i. We constrain the possible relationships between these rates such that the transmission matrix $\beta_{\mathrm{ij}}$ may be written as an outer product ($\beta_{\mathrm{ij}}=x_{i}y_{j}$) of two vectors, ${(x}_{1},\cdots ,x_{n})$ and $\left(y_{1},\cdots ,y_{n}\right)$. These vectors represent age-specific parameters for susceptible and infected individuals, respectively. This assumption is made for ease of derivation, and its implications are treated in the discussion. The model thus has 2n degrees of freedom wrt. infection rates. We assume a constant total population size and that there is no maternal–fetal transmission of disease or immunity, such that everyone is born susceptible. Thereby, the equation for the susceptible individuals in the first age stage becomes

[2] $$\begin{array}{c} \frac{dS_{1}}{\mathrm{dt}}=\sum_{i=1}^{n}\left(d_{i}I_{i}+\delta_{i}\left(S_{i}+I_{i}+R_{i}\right)\right)\\ \;-{(a}_{1}+\delta_{1})S_{1}+\omega R_{1}-\sum_{i=1}^{n}\beta_{1i}S_{1}I_{i}. \end{array}$$

and the equations for the infected and recovered in the first age stage are given by Eq. **1** with i=1.

A fundamental quantity for the model shown in Eq. **1** is the basic reproduction number $R_{0}$, the mean number of individuals infected by a single infectious individual in a completely susceptible population. Mathematically, $R_{0}$ is the dominant eigenvalue of the next-generation matrix associated with the linearization at the disease-free equilibrium (23, 24). When transmission is supercritical ($R_{0}>1$), an infection may go from being initially rare to becoming epidemic. More precisely, an $R_{0}>1$ ensures a positive outbreak probability in a sufficiently large population, although this probability may be too small to be practically meaningful. Backward bifurcations may mathematically allow disease to persist despite an $R_{0}<1$ in rare situations (25) that appear not to occur under our mathematical framework. If an epidemic outbreak occurs and the susceptible population is replenished over time, it may later become endemic (26). If we neglect disease-related mortality, which is reasonable in the low case-fatality rate (CFR) case, eventually the endemic equilibrium for the aggregate compartments is

$S^{*}=\frac{1}{R_{0}},I^{*}=\frac{\delta \left(1-\frac{1}{R_{0}}\right)}{\gamma +\delta -\frac{\omega \gamma }{\omega +\delta }},and\;R^{*}=\gamma \frac{I^{*}}{\omega +\delta }$, where $S^{*},I^{*},R^{*}$ here refer to the total susceptible, infected, and recovered populations, respectively, and δ and γ are averaged population level rates as weighted by age structure.

### Evolutionary Stability in the Extended Demographic Epidemiology Model.

Following Reed and Stenseth (12), in order to assess the invasion ability of a potentially invading variant (Fig. 1*B*), we extend the above model to incorporate mutant variants. Accordingly, ${\tilde{I}}_{i}$ is the number of infections that are induced by the mutant strain in age stage i. In general terms, the mutant strain may have different infectivities between age stages, i.e., a different ${\tilde{\beta }}_{\mathrm{ij}}$ matrix and recovery rates ${\tilde{\gamma }}_{i}$, as compared with the wild-type strain. We believe the age dependence of $\gamma_{i}$ to be a useful assumption, since the interaction between host cells and immune systems on one side and pathogens on the other is shaped by host age, at least in mammals (27–29). For example, T cell diversity and function are well known to degrade with age (30, 31). This age dependence is likely to exert an evolutionary pressure on the pathogen. Interestingly, age-dependent evolution has also been observed in plant viruses (32) and an evolutionary pressure in the opposite direction, exerted by viruses on human aging, has been suggested (33).

For clarity, we assume that the disease mortality, loss of immunity, and aging rate are the same for both the original and novel variant. We extend the model of Eq. **1** as follows (34):

[3] $$\begin{array}{c} \begin{array}{c} \frac{dS_{i}}{\mathrm{dt}}=a_{i-1}S_{i-1}-a_{i}S_{i}-\delta_{i}S_{i}+\omega {(R}_{i}+{\tilde{R}}_{i})-\sum_{j}\beta_{\mathrm{ij}}S_{i}I_{j}\\ \;-\underset{\mathrm{mutant infection}}{\underset{︸}{\sum_{j}{\tilde{\beta }}_{\mathrm{ij}}S_{i}{\tilde{I}}_{j}}}\\ \frac{dI_{i}}{\mathrm{dt}}=a_{i-1}I_{i-1}+\sum_{j}\beta_{\mathrm{ij}}S_{i}I_{j}-\left(a_{i}+\gamma_{i}+d_{i}+\delta_{i}\right)I_{i}\\ \begin{array}{c} \frac{dR_{i}}{\mathrm{dt}}=a_{i-1}R_{i-1}+\gamma_{i}I_{i}-{(a}_{i}+\delta_{i}+\omega )R_{i}\\ \frac{d{\tilde{I}}_{i}}{\mathrm{dt}}=a_{i-1}{\tilde{I}}_{i-1}+\underset{\mathrm{mutant infection}}{\underset{︸}{\sum_{j}{\tilde{\beta }}_{\mathrm{ij}}S_{i}{\tilde{I}}_{j}}}-\left(a_{i}+{\tilde{\gamma }}_{i}+d_{i}+\delta_{i}\right){\tilde{I}}_{i}\\ \frac{d{\tilde{R}}_{i}}{\mathrm{dt}}=a_{i-1}{\tilde{R}}_{i-1}+\underset{\mathrm{mutant recovery}}{\underset{︸}{{\tilde{\gamma }}_{i}{\tilde{I}}_{i}}}-{(a}_{i}+\delta_{i}+\omega ){\tilde{R}}_{i}. \end{array} \end{array} \end{array}$$

When the mutant is at low densities, its dynamics can be approximated by the linear system of equations:

[4] $$\begin{array}{c} \frac{d{\tilde{I}}_{i}}{\mathrm{dt}}=a_{i-1}{\tilde{I}}_{i-1}+\underset{\mathrm{mutant infection}}{\underset{︸}{\sum_{j}{\tilde{\beta }}_{\mathrm{ij}}S_{i}^{*}{\tilde{I}}_{j}}}-\left(a_{i}+{\tilde{\gamma }}_{i}+d_{i}+\delta_{i}\right){\tilde{I}}_{i}\\ \frac{d{\tilde{R}}_{i}}{\mathrm{dt}}=a_{i-1}{\tilde{R}}_{i-1}+\underset{\mathrm{mutant recovery}}{\underset{︸}{{\tilde{\gamma }}_{i}{\tilde{I}}_{i}}}-(a_{i}+\delta_{i}+\omega ){\tilde{R}}_{i}. \end{array}$$

The ability of a mutant to invade is determined by its invasion number $Q_{0}$, the mean number of susceptibles infected by an individual carrying the mutant strain when the populations are at the endemic equilibrium $\left(S_{i}^{*},I_{i}^{*},R_{i}^{*}\right)$ of the resident strain. Analogously to $R_{0}$, $Q_{0}$ corresponds to the dominant eigenvalue of the next-generation operator of the linearized mutant dynamics Eq. **4**. If the invasion number of the mutant is less than one, it fails to invade. Hence, the resident strain corresponds to an ESS when $Q_{0}$ is less than one for all competing mutant life histories.

### Deriving the ESS.

The age-structured epidemiological model Eq. **3** can be used to investigate the ESS of an evolving pathogen. We assume that the age-specific infectivity is a function of some evolutionary parameter(s) which may vary with age. For the purposes of this paper, we will apply a variant of the trade-off hypothesis (4), that is, we assume a trade-off between the infectivity of the disease (β) and its recovery rate (γ) such that β=β(γ). To ensure an ESS exists, we assume that $\beta \left(\gamma \right)$ is a concave function. We further assume that the transmission matrix element $\beta_{\mathrm{ij}}$ only depends on $\gamma_{j}$. This is reasonable as infectivity depends on pathogen shedding, which in turn mostly depends on the interaction between the pathogen and the infected individual, here of age stage j (16, 35–37). Finally, we assume that aging is slow relative to epidemic dynamics as is the case for acute human infections.

Given all these assumptions, one can show that the ESS must satisfy the relation

[5] $$\beta_{\mathrm{ii}}\prime \left(\gamma_{i}\right)\cdot \left(a_{i}+\gamma_{i}+d_{i}+\delta_{i}\right)=\beta_{\mathrm{ii}}\left(\gamma_{i}\right).$$

This is demonstrated in the methods section (see also Eq. **11**).

**Fig. 2.**
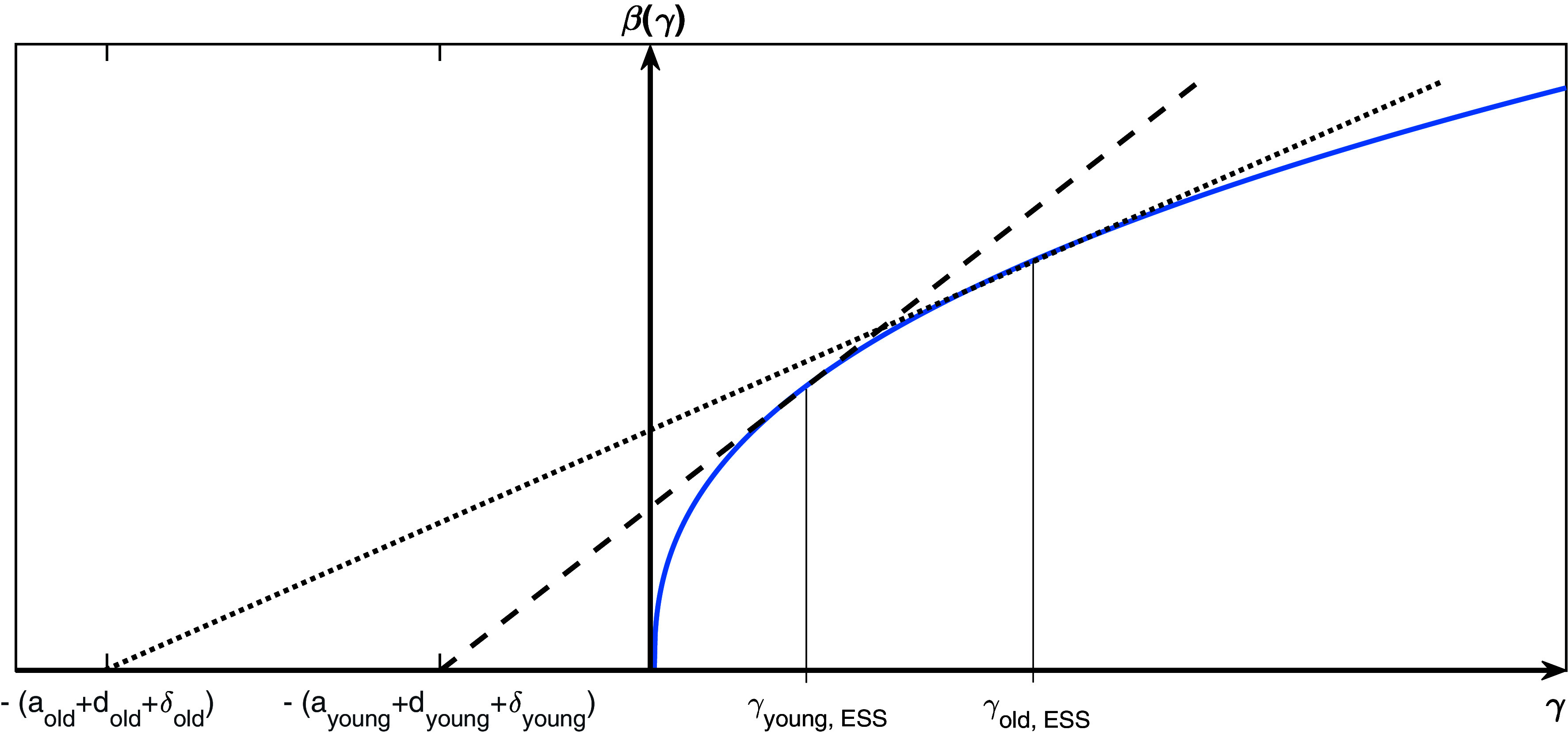
An illustration of Eq. **5**. Solving the set of equations is equivalent to finding the point where a line through $-(a_{i}+\delta_{i}+d_{i})$ is tangent to the trade-off function $\beta (\gamma_{i})$, as demonstrated in Ref. 5. The higher the background and disease mortalities are, the higher the value of the recovery rate, $\gamma_{i}$, and infectivity, β, will be at the ESS.

If $\beta_{\mathrm{ii}}\left(\gamma_{i}\right)$ are concave functions, we can always find the ESS by solving Eq. **5** for all age stages. This is a generalization of a well-known result from evolutionary biology to a model with multiple age stages (2). Fig. 2 illustrates this result geometrically. The figure shows that increasing background mortality in a given age stage results in selection for increased infectivity in that stage as well as the recovery rate γ increasing. More succinctly, the disease is predicted to evolve toward greater acuteness.

Running an ODE simulation of this model, where we let new randomly generated variants invade and replace the resident variants, invaders with higher $R_{0}$ will tend to win over time, at least in the illustrative case where β has the form $\beta \propto \gamma^{p}$. The numerical simulations thus confirm that $R_{0}$ is expected to be maximized over the course of evolution. Our numerical work on the topic can be found in supplement. The conditions for $R_{0}$ maximization have been studied extensively by Srestha and King et al. (13, 38), and the role of $R_{0}$ in pathogen invasion is further elucidated by Bjørnstad (39).

## Discussion

The variant replacement waves of SARS-CoV-2 have illustrated the importance of applying fundamental evolutionary ideas such as the ESS to anticipate likely disease characteristics during the pandemic and endemic stages of the emergence of a novel pathogen. In relation to this, the evolutionarily optimal length of the presymptomatic infectious period of COVID-19 and similar diseases has previously been examined (40, 41). As illustrated by the current pandemic, the disease burdens of many human pathogens, including SARS-CoV-2, influenza viruses, and childhood infections, are highly stratified by age. Various previous studies have shown that the age pyramid and social interactions between age stages may strongly shape disease dynamics and the public health burden (42–48). Therefore, it is critical to explicitly investigate the likely evolutionary trajectories of epidemic diseases within an age-structured context. To this end, we have here provided a theoretical framework.

In our model, the age-specific rates of infectivity ($\beta_{\mathrm{ij}})$ and recovery ($\gamma_{i})$ at the ESS are associated with the corresponding disease fatality rate and the background mortality as demonstrated in Eq. **5**. By characterizing the ESS using age-specific $\beta_{\mathrm{ii}}\left(\gamma_{i}\right)$, our study shows that $\beta_{\mathrm{ii}}\left(\gamma_{i}\right)$ being concave functions is a sufficient condition for an ESS to exist. That is, the disease should gain progressively less in infectivity as it becomes more acute. Otherwise, it would be possible for γ=0 or γ=∞ to be optimal for the disease, and the model might predict the evolution of a biologically impossible pathogen that is infinitely infectious for infinite time—a so-called Darwinian demon.

Our results are somewhat limited in generality by the assumptions made in the model. Of these, the assumptions that variants cause total cross-immunity to each other, that case fatality rates be low enough to not affect demographics, and that the infection matrix be an outer product of two vectors ($\beta_{\mathrm{ij}}=x_{i}y_{j}$) are particularly important. The low CFR assumption holds well for several human respiratory pathogens [prominently seasonal influenza (49) and COVID-19 (50)] but is certainly not universal, especially not if we turn to pathogens of other species. The cross-immunity assumption likewise holds for some pathogens but not others (51, 52). Finally, the outer product assumption for the infection matrix is likely to hold if the probability of socializing and of infection between any pair of individuals is given roughly by a product of each individual’s independent propensity to socialize and infect. In practice, however, the social structure of human societies is more complex than that. To quantify the effect of the former two caveats, we numerically simulate variant replacements in cases where disease CFRs are much higher than background death rates, and where $\beta_{\mathrm{ij}}=c_{\mathrm{ij}}\cdot \beta \left(\gamma_{j}\right)\neq x_{i}y_{j}$ with $c_{\mathrm{ij}}$ being a random matrix with entries between 0 and 1. Breaking these assumptions turn out to have a limited effect on the overall direction of evolution (*SI Appendix*). The lacking cross-immunity on the other hand would require larger alterations to the model which we feel would put it beyond the scope of this study. This of course means that our results might not apply to pathogens with limited cross-immunity between strains.

Our results show that a higher background or disease-related mortality rate in a given age stage would favor a higher β and consequently in the case of a convex β(γ) function a higher γ (i.e., shorter duration of infection) at the ESS. This means that evolution would result in variants that are more infectious for any age stages with high CFR or higher background mortality, whereas evolution would lower the infection rate for age stages with a low CFR or background mortality (Fig. 2). Based on this, we would expect successful new variants to be slow to infect but long lived in younger age groups, and fast but short lived in older age groups.

Our present findings are, from the perspective of evolutionary strategy, consistent with the severity of the disease increasing in proportion with the age of the host. We predict that the infectious period will be shorter, but the infectivity (and thus likely the pathogen load) higher among people with a higher background mortality. From this, we might expect the risk of death from the disease to evolve toward an even greater age dependence than what is expected from immune senescence in the elderly. On the other hand, the maximum $R_{0}$ will for some realistic assumptions about $\beta \left(\gamma \right)$ be higher among young people due to their lower background mortality. If $\beta \left(\gamma \right)$ once again takes a power law form $\beta \left(\gamma \right)\propto \gamma^{p}$ where p<1, $R_{0}\approx \frac{\beta \left(\gamma \right)}{\gamma +\delta +d}$ will be a decreasing function of γ. This would suggest that young people are inherently more important contributors to the spread of an epidemic than older people, at least if it evolves toward an endemic age-specific ESS state.

SARS-CoV-2 originally sparked our interest in this topic, but whether its evolution supports our hypotheses is not clear. Seemingly consistent with our findings, previous studies have shown substantial numbers of asymptomatic (but still infectious) COVID-19 cases in young people (53, 54). Also, younger people were important drivers of the pandemic, although this was more likely due to social factors than to an inherently higher transmissibility of the variants in this group (55, 56). A study has suggested that COVID-19 patients with risk factors such as high age or obesity shed more virus aerosols and are thus more likely to be superspreaders (57). This has, however, been the case from a very early stage in the pandemic, indicating that it is less likely to be an adaptation to human hosts. As a further reason for caution, a different study demonstrates that older patients and those with higher viral load have a slower viral clearance and thus might remain infectious for a longer time, contradicting our results (58). From the outset, the pathogen was more virulent in older populations. As it turns out, this may well not correlate with a shorter infectious period, at least as measured by the decline rate of viral load. At this point, we are thus unable to conclude whether SARS-CoV-2 evolution follows the path outlined by our hypothesis.

To summarize, our results highlight the importance of age-based social structure and varying background mortality for pathogen evolution. Our results are sufficiently general to be applicable to a variety of pathogens that spread in societies with diverse social structures.

## Conclusion

In this contribution, we have derived conditions for an ESS for a pathogen spreading in age-structured populations. We show how the likely evolutionary endpoint depends on background mortality and disease fatality rates across the age groups of a population. Evolutionarily, groups with higher mortalities are predicted to end up with shorter, more intense infectious periods given a trade-off between disease infectivity and duration. Our findings could help predict the epievolutionary dynamics of pandemics as they reach endemicity.

## Materials and Methods

The reproductive number for each variant of the pathogen in our model can be calculated as the largest eigenvalue of their next-generation matrices (25). For simplicity, we assume that age-specific characteristics of the susceptible individuals and the infected individuals contribute multiplicatively to the infection rates, i.e., $\beta_{\mathrm{ij}}=x_{i}y_{j}$. With this assumption, the reproductive number is the trace of the next-generation matrix.

***Proof:*** Let ${(\hat{S}}_{1},\cdots ,{\hat{S}}_{n})$ be the vector of the densities of the susceptible at the disease-free equilibrium of Eq. **1**. The entries of this vector are given by


$${\hat{S}}_{1}=\frac{S_{0}}{1+\sum_{k=2}^{n}\alpha_{k}},$$

$${\hat{S}}_{i}=\frac{\alpha_{i}S_{0}}{1+\sum_{k=2}^{n}\alpha_{k}}for\;i\geq 2,$$

where

$$\alpha_{i}=\prod_{k=2}^{i}\frac{a_{k-1}}{a_{k}+\delta_{k}}.$$

The linearization of Eq. **1**;at the disease-free equilibrium can be written as a continuous-time matrix model

$$\frac{\mathrm{dI}}{\mathrm{dt}}=\left(T+\Sigma \right)I,$$

where $I=(I_{1},\cdots ,I_{n})$ is the vector of infected densities, T is the transmission matrix with entries $T_{\mathrm{ij}}=\beta_{\mathrm{ij}}{\hat{S}}_{i}$, and Σ is the transition matrix given by

$$\Sigma =\left[\begin{array}{ccccc} -\mu_{1} & 0 & \cdots & 0 & 0\\ a_{1} & -\mu_{2} & 0 & \cdots & 0\\ 0 & a_{2} & \ddots & \ddots & \vdots \\ \vdots & \ddots & \ddots & \ddots & 0\\ 0 & \cdots & 0 & a_{n-1} & -\mu_{n} \end{array}\right],$$

where $\mu_{i}=a_{i}+\delta_{i}+\gamma_{i}+d_{i}$. The next-generation matrix [see, e.g., Diekmann et al. (59)] is $-T\Sigma^{-1}$, where the inverse of the transition matrix equals

$$\Sigma^{-1}=\left[\begin{array}{cccc} -\frac{1}{\mu_{1}} & 0 & \cdots & 0\\ -\frac{a_{1}}{\mu_{1}\mu_{2}} & -\frac{1}{\mu_{2}} & \ddots & \vdots \\ \vdots & \ddots & \ddots & 0\\ -\frac{a_{1}\cdots a_{n-1}}{\mu_{1}\cdots \mu_{n}} & \cdots & -\frac{a_{n-1}}{\mu_{n-1}\mu_{n}} & -\frac{1}{\mu_{n}} \end{array}\right],$$

If $\beta_{\mathrm{ij}}=x_{i}y_{j}$, then T is an outer product of the vectors $\left(x_{1}{\hat{S}}_{1},\cdots ,x_{n}{\hat{S}}_{n}\right)$ and $\left(y_{1},\cdots ,y_{n}\right)$, and consequently T is a rank 1 matrix. As $\Sigma^{-1}$ is invertible, the next-generation matrix $-T\Sigma^{-1}$ is also rank 1. As the trace of a matrix is the sum of the eigenvalues and $-T\Sigma^{-1}$ is rank 1, the trace of $-T\Sigma^{-1}$ equals the unique positive eigenvalue of $-T\Sigma^{-1}$, i.e., $R_{0}$. ■

Computing this trace, we get

[6] $$R_{0}=\sum_{j=1}^{n}\sum_{i=1}^{j}\frac{\beta_{\mathrm{ij}}\left({\tilde{\gamma }}_{j}\right){\hat{S}}_{i}\prod_{k=i}^{j-1}a_{k}}{\prod_{k=i}^{j}\left(a_{k}+\gamma_{k}+d_{k}+\delta_{k}\right)}.$$

Similarly, we can derive the invasion number of the invader as

[7] $$Q_{0}=\sum_{j=1}^{n}\sum_{i=1}^{j}\frac{\beta_{\mathrm{ij}}\left({\tilde{\gamma }}_{j}\right)S_{i}^{*}\left(\gamma_{i}\right)\prod_{k=i}^{j-1}a_{k}}{\prod_{k=i}^{j}\left(a_{k}+{\tilde{\gamma }}_{k}+d_{k}+\delta_{k}\right)},$$

where $S_{i}^{*}$ are the susceptible populations at the endemic state of the wild-type strain. The ESS occurs when $Q_{0}<1$ for all $\tilde{\gamma }\neq \gamma$. At the ESS, no invader can gain an advantage over the circulating strain by changing γ. It is therefore a necessary condition that

$$\nabla_{\tilde{\gamma }}Q_{0}{|}_{\tilde{\gamma }=\gamma }=0.$$

All terms that do not depend on ${\tilde{\gamma }}_{l}$ vanish when taking the derivative with respect to ${\tilde{\gamma }}_{l}$, meaning that only terms where j≥l remain. We can write n equations

[8] $$\frac{\partial }{\partial {\tilde{\gamma }}_{l}}\sum_{j=l}^{n}\sum_{i=1}^{j}\frac{\beta_{\mathrm{ij}}\left({\tilde{\gamma }}_{j}\right)S_{i}^{*}\left(\gamma_{i}\right)\prod_{k=i}^{j-1}a_{k}}{\prod_{k=i}^{j}\left(a_{k}+{\tilde{\gamma }}_{k}+d_{k}+\delta_{k}\right)}{|}_{\tilde{\gamma }=\gamma }=0.$$

Infectivity ($\beta =\beta \left(\gamma \right)\;and\;\tilde{\beta }=\beta \left(\tilde{\gamma }\right)$) and recovery rate ($\gamma \;and\;\tilde{\gamma }$) differ between the wild-type and invading variant. The terms of this sum all contain a number of factors of the form $\frac{1}{a_{j}+\gamma_{j}+d_{j}+\delta_{j}}\cdot \frac{a_{j-1}}{a_{j-1}+\gamma_{j-1}+d_{j-1}+\delta_{j-1}}\cdots \frac{a_{i}}{a_{i}+\gamma_{i}+d_{i}+\delta_{i}}$. Assuming that the disease dynamics happen on a much faster timescale than aging, any term with i≠j—which contains multiple such factors—will be much smaller than 1. To be more precise, if recovery or death happens on a timescale of weeks, while aging happens on a timescale of years, the corresponding rates $\gamma_{i}+d_{i}$ will be of the order 50 times larger than $a_{i}$. If a disease evolves to be very long-lasting, and we separate the population into n≫1 age stages, this assumption may ultimately break down. Nonetheless, this seems to be a very specific case, and our numerical simulations do not show such a breakdown occurring. We can therefore neglect these terms and write the above equations as

[9] $$\frac{\partial }{\partial {\tilde{\gamma }}_{l}}\frac{\beta_{ii}\left({\tilde{\gamma }}_{i}\right)S_{i}^{*}(\gamma_{i})}{\left(a_{i}+{\tilde{\gamma }}_{i}+d_{i}+\delta_{i}\right)}{|}_{\tilde{\gamma }=\gamma }=0,$$

additionally making use of the fact that the derivative wrt. ${\tilde{\gamma }}_{l}$ of any term where i≠l will be zero. Calculating the derivatives

[10] $$\begin{array}{c} \frac{\partial }{\partial {\tilde{\gamma }}_{i}}\frac{\beta_{\mathrm{ii}}\left({\tilde{\gamma }}_{i}\right)S_{i}^{*}\left(\gamma_{i}\right)}{\left(a_{i}+{\tilde{\gamma }}_{i}+d_{i}+\delta_{i}\right)}{|}_{\tilde{\gamma }=\gamma }\\ \;=\frac{\beta_{\mathrm{ii}}\prime \left({\tilde{\gamma }}_{i}\right)S_{i}^{*}\left(\gamma_{i}\right)\left(a_{i}+{\tilde{\gamma }}_{i}+d_{i}+\delta_{i}\right)-\beta_{\mathrm{ii}}\left({\tilde{\gamma }}_{i}\right)S_{i}^{*}\left(\gamma_{i}\right)}{{\left(a_{i}+{\tilde{\gamma }}_{i}+d_{i}+\delta_{i}\right)}^{2}}{|}_{\tilde{\gamma }=\gamma }, \end{array}$$

and setting this to zero, we arrive at

[11] $$\beta_{\mathrm{ii}}\prime \left(\gamma_{i}\right)S_{i}^{*}\left(\gamma_{i}\right)\left(a_{i}+\gamma_{i}+d_{i}+\delta_{i}\right)-\beta_{\mathrm{ii}}\left(\gamma_{i}\right)S_{i}^{*}\left(\gamma_{i}\right)=0.$$

Since $S_{i}^{*}\left(\gamma_{i}\right)\neq 0$, this implies that $\beta_{\mathrm{ii}}\prime \left(\gamma_{i}\right)\left(a_{i}+\gamma_{i}+d_{i}+\delta_{i}\right)-\beta_{\mathrm{ii}}\left(\gamma_{i}\right)=0$. We thus recover Eq. **5**:

$$\beta_{\mathrm{ii}}\prime \left(\gamma_{i}\right)\cdot \left(a_{i}+\gamma_{i}+d_{i}+\delta_{i}\right)=\beta_{\mathrm{ii}}\left(\gamma_{i}\right),$$

If we assume that the infection rate from class i to j is proportional to some pairwise contact rate between these classes, multiplied by an infectivity function $\beta \left(\gamma_{i}\right)$, we may write $\beta_{\mathrm{ii}}=c_{\mathrm{ii}}\beta \left(\gamma_{i}\right)$ where $c_{\mathrm{ii}}$ is the contact rate within class i. We now see that the contact rate, $c_{\mathrm{ii}}$, cancels out:

[12] $$\beta_{\mathrm{ii}}\prime \left(\gamma_{i}\right)\cdot \left(a_{i}+\gamma_{i}+d_{i}+\delta_{i}\right)=\beta \left(\gamma_{i}\right).$$

This means that if we can write the elements of $\beta_{\mathrm{ij}}$ as consisting of a contact rate multiplied by an infectivity trade-off function, the exact contact rates are not important in determining the ESS of the pathogen. Rather, what determines the ESS will be the form of the infectivity function β(γ).

Since we have made several assumptions in order to arrive at the main result of Eq. **5**, we test how applicable these results are to a general system of equations by creating an ODE-based numerical simulation of evolution. The model simulates the populations of a resident strain and an invader and allows the invader to replace the resident if its population gets much larger.

The assumption about the structure of $\beta_{\mathrm{ij}}$ as an outer product $x_{i}y_{j}$ is a problematic point for our analysis and deserves additional treatment. It only holds in practice if the contact rate between two groups is the product of some natural propensity to socialize of each of those groups, which is at best only approximately true for human age groups (60). However, we have analyzed the sensitivity of our results to this assumption. This sensitivity analysis shows that breaking the assumption by simply multiplying each element in $\beta_{\mathrm{ij}}$ by a random number does not significantly affect the conclusion that $R_{0}$ tends to grow over time, as is often (wrongly) assumed to be the usual case (14, 61). Finally, the numerical work suggests that values of γ in the simulation decrease toward those given by the Eq. **5**, although the evolutionary dynamics slow down so much over the course of these simulations that the resultant values of γ never truly approach the predicted ESS values. The results of this numerical work can be found in supplementary material, along with a more detailed description of the algorithm used. The script used for the simulations can be found on Figshare (10.6084/m9.figshare.26798527.v2). These numerical results show that it is reasonable to assume that the pathogen will evolve toward maximizing $R_{0}$.

## Supplementary Material

Appendix 01 (PDF)

## Data Availability

Numerical code data have been deposited in Figshare (https://doi.org/10.6084/m9.figshare.26798527.v2) (62).
