# Supplementary material for: Epidemic evolutionarily stable strategies within an age-structured host population
Source: Proc Natl Acad Sci U S A. 2025 Mar 18;122(12):e2418170122. doi: 10.1073/pnas.2418170122 (PMC11962425; doi:10.1073/pnas.2418170122)
Supplement: Supplementary file 1 — Appendix 01 (PDF) [file pnas.2418170122.sapp.pdf]

Supplementary material to "Epidemic Evolutionarily Stable Strategies (ESS) within an age-structured host population" by Andreas Eilersen, Ottar N. Bjørnstad, Ruiyun Li, Sebastian J. Schreiber, Zeyuan Pei, & Nils Chr. Stenseth

Simulating the system

We here aim at assessing whether a pathogen spreading in a society like that of the main article will always evolve towards a higher R_0. To this end, we implement variant invasion and replacement in the model. Then, we calculate the R_0 for each successful invader by using the next-generation matrix method and plugging in the populations of all age stages at the disease-free equilibrium.

Evolution is here simulated by introducing a new variant with random γ_i values into the system at a very low population. We then solve the system of equations until the new variant either dies out or starts taking over the system. If it comes to dominate the population of infected ($\widetilde{I} \geq 4I$), we let it take on the role of resident strain. We then replace the former resident with a new strain with some random mutations in γ_i. This process is iterated over an evolutionary timescale, in this case $2 \cdot 10^7$ time units. Time units roughly correspond to days, given that we use per-day background death rates. We fix these death rates for each age stage based on the National Vital Statistics Reports life table [i] (see fig. S5). The immunity loss rate ω is fixed at $\omega = 0.005\ time\ units^{-1}$. The code used to perform these simulations can be found on Figshare (DOI: 10.6084/m9.figshare.26798527.v2).

Results – evolution over time

As mentioned above, we run the evolutionary simulation outlined here for $T = 2 \cdot 10^7$ time units. This allows taking note of the behavior of the infectious population and the γ parameters over time.

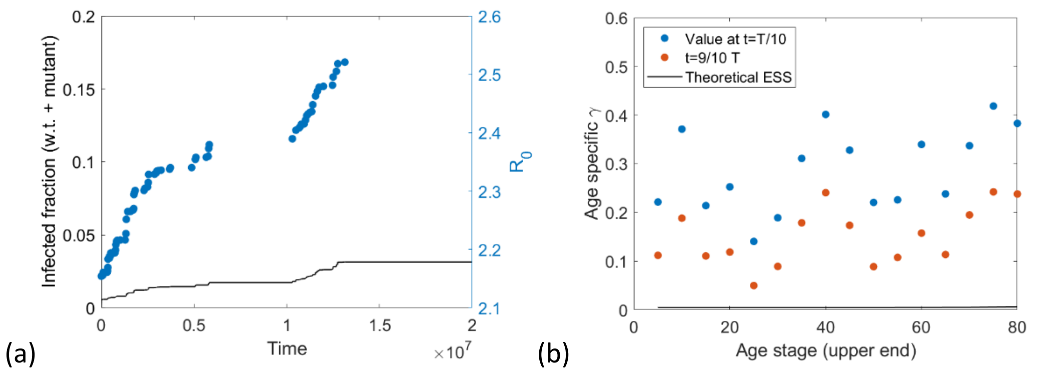

Fig. S1: *Plots of (a) the infected fraction over an evolutionary timescale and (b) mean γ_i-values early in the simulation ($t = T/10$) and later ($t = \frac{9}{10}T$) over five runs of the simulation with duration $T = 2 \cdot 10^7$. It can be seen that evolution here tends to increase both the steady state infected population and the value of R_0 with each variant replacement. The values of γ mostly decrease over time, though the approach slows down well before the predicted ESS. The predicted*

ESS values of γ *are all very low (of the order* $5 \cdot 10^{-3}$*), and hence the difference between age stages can be hard to see.* $\beta_{ij} = 2\gamma_j^{0.9}$.

In fig. S1 we see two plots, panel (a) showing a single run of the simulation with duration $T = 2 \cdot 10^7$ time units (line, left y-axis) along with calculated R_0 values of predominant variants over time (blue dots, right y-axis). Here, we see that variants are quickly replaced in the beginning, but more and more slowly as more optimal variants become established. Confirming our expectations, R_0 increases with each variant replacement. Panel (b) shows the γ-values early and late in the same simulation, averaged over five runs. For comparison, the predicted ESS values ($\gamma_i^* = (a_i + d_i + \delta_i) \cdot \frac{p}{1-p}$ where p is the power of γ in the expression for β_{ij}, that is $\beta_{ij} = A \cdot \gamma_j^p$) are also plotted. We use the values $A = 2$ and $p = 0.9$. We observe that γ drops toward the predicted ESS over the course of the simulation, but that this drop slows down and stops before the γ-values approach the ESS. We believe that the slowdown of invasion dynamics observed in panel (a) is the cause for this discrepancy.

Testing the effects of breaking the assumptions made in the main manuscript, we generally observe that this does not affect the overall dynamics of evolution. Successive variant replacements lead to increasing R_0, and γ-values that decrease towards the theoretically predicted ESS values, albeit slowing to a halt well before they are reached. In fig. S2, we see the results of breaking the outer product assumption. This is done by multiplying β_{ij} element-wise by some random matrix c_{ij} with entries between 0 and 1. These results overall look highly similar to fig. S1. To achieve an initial R_0 greater than 1, we had to increase the proportionality constant relating β_{ij} to γ_j, such that $\beta_{ij} = 3\gamma^{0.9}$ instead of $\beta_{ij} = 2\gamma^{0.9}$ as in the other figures.

Fig. S3 shows the same plots, but where the population is no longer assumed constant. Instead, disease mortality can now deplete the population. To roughly model population regrowth a logistic growth term, $r_{growth} \cdot N \cdot (1 - N)$, is added to the equation for S_1 (since everyone is born susceptible). Here, N is the sum of all populations and $r_{growth} = \frac{1}{5 \cdot 365}\ time\ units^{-1}$ is a very rough estimate of a maximum population growth rate after a depletion event. These changes also have little effect on the evolutionary dynamics as long as disease CFR is small, of the order 1-10 % for most γ values. Larger disease death rates (d_i) end up dominating the theoretically predicted ESS ($\gamma_i^* = (a_i + d_i + \delta_i) \cdot \frac{p}{1-p}$). If death rates are large, the predicted γ_i^*'s at the ESS also all become large, with a small relative difference between them. At high CFR's we therefore observe that the initial strain remains indefinitely (see fig. S4). We do not see variant replacements when the predicted ESS values of γ_i^* are greater than the starting values.

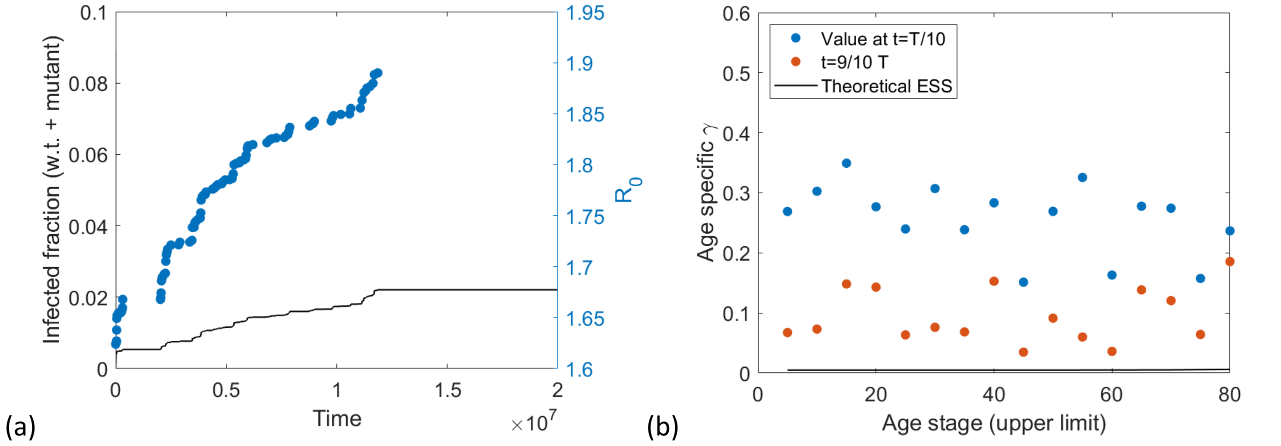

Fig. S2: *(a) Infected fraction of population (left y-axis, black line) and R_0 values (right y-axis, blue dots) when breaking the outer product assumption ($\beta_{ij} = x_i y_j$). (b) Mean γ_i-values early in the simulation ($t = T/10$) and later ($t = \frac{9}{10}T$) over five runs of the simulation with duration $T = 2 \cdot 10^7$. We see the same tendency towards increasing R_0 and decreasing γ_i's as in fig. S1. $\beta_{ij} = 3c_{ij}\gamma_j^{0.9}$.*

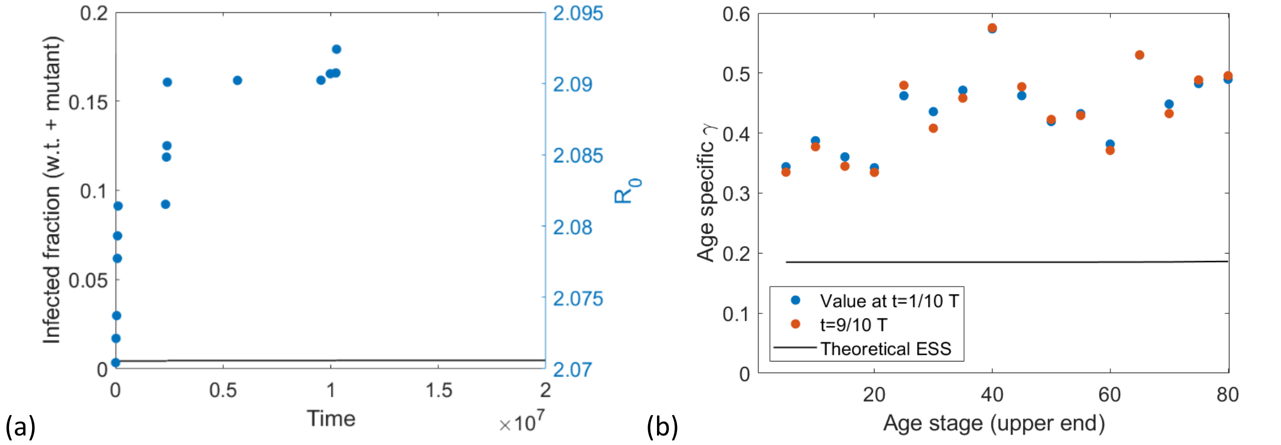

Fig. S3: *(a) Infected fraction of population (left y-axis, black line) and R_0 values (right y-axis, blue dots) when breaking the assumption of constant population (b) Mean γ_i-values early in the simulation ($t = T/10$) and later ($t = \frac{9}{10}T$) over five runs of the simulation with duration $T = 2 \cdot 10^7$. We again see the same tendency towards increasing R_0 and decreasing γ_i's. $\beta_{ij} = 2\gamma_j^{0.9}$; disease death rate $d_i = 0.02$.*

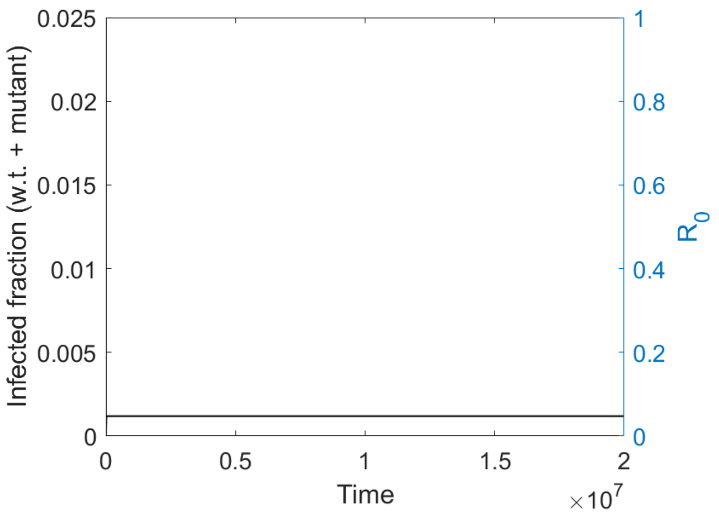

Fig. S4: *Infected fraction of population (black line, note the smaller scale of y-axis) if disease death rate is high ($d = 0.1$ per unit time of illness for all ages). R_0 of invading variants would be shown too, but no invasions occur and the initial variant remains indefinitely.* $\beta_{ij} = 2\gamma_j^{0.9}$.

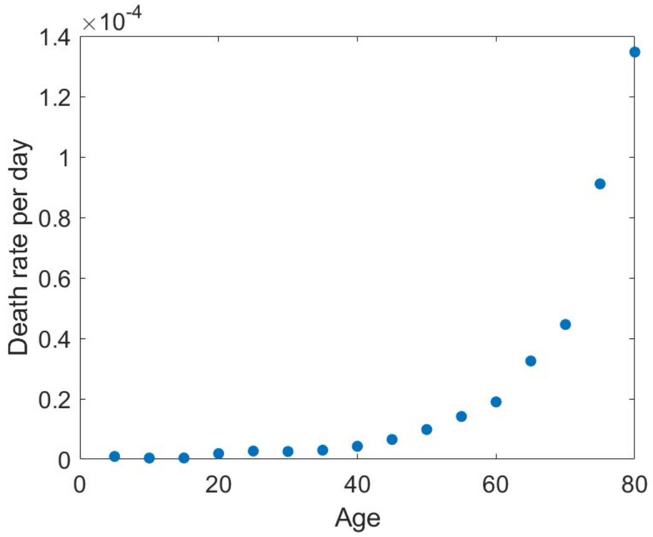

Fig. S5: *Daily background death rates δ_i used in these simulations, included for reference. Statistic from Ref.* [i].

---

[i] *Life table for the total population: United States, 2003*. National Vital Statistics Reports, Vol. 54 (14), 2006
